# Change-Related Acceleration Effects on Auditory Steady State Response

**DOI:** 10.3389/fnsys.2019.00053

**Published:** 2019-10-15

**Authors:** Shunsuke Sugiyama, Tomoaki Kinukawa, Nobuyuki Takeuchi, Makoto Nishihara, Toshiki Shioiri, Koji Inui

**Affiliations:** ^1^Department of Psychiatry and Psychotherapy, Graduate School of Medicine, Gifu University, Gifu, Japan; ^2^Department of Anesthesiology, Graduate School of Medicine, Nagoya University, Nagoya, Japan; ^3^Department of Psychiatry, Aichi Medical University, Nagakute, Japan; ^4^Multidisciplinary Pain Center, Aichi Medical University, Nagakute, Japan; ^5^Institute for Developmental Research, Aichi Human Service Center, Kasugai, Japan; ^6^Department of Integrative Physiology, National Institute for Physiological Sciences, Okazaki, Japan

**Keywords:** ASSR, change-related response, latency, MEG, phase resetting

## Abstract

Rapid detection of sensory changes is important for survival. We have previously used change-related cortical responses to study the change detection system and found that the generation of a change-related response was based on sensory memory and comparison processes. However, it remains unclear whether change-related cortical responses reflect processing speed. In the present study, we simultaneously recorded the auditory steady-state response (ASSR) and change-related response using magnetoencephalography to investigate the acceleration effects of sensory change events. Overall, 13 healthy human subjects (four females and nine males) completed an oddball paradigm with a sudden change in sound pressure used as the test stimulus, i.e., the control stimulus was a train of 25-ms pure tones at 75 dB for 1,200 ms, whereas the 29th sound at 700 ms of the test stimulus was replaced with a 90-dB tone. Thereafter, we compared the latency of ASSR among four probabilities of test stimulus (0, 25, 75, and 100%). For both the control and test stimulus, stronger effects of acceleration on ASSR were observed when the stimulus was rarer. This finding indicates that ASSR and change-related cortical response depend on physical changes as well as sensory memory and comparison processes. ASSR was modulated without changes in peripheral inputs, and brain areas higher than the primary cortex could be involved in exerting acceleration effects. Furthermore, the reduced latency of ASSR clearly indicated that a new sensory event increased the speed of ongoing sensory processing. Therefore, changes in the latency of ASSR are a sensitive index of accelerated processing.

## Introduction

For survival, rapid detection of changes in the sensory environment is essential. Therefore, one of the most important functions of sensory systems is the detection of changes. Neural networks sensitive to sensory changes have been identified in humans (Downar et al., 2000; Tanaka et al., 2009b). Because a change detection system spontaneously operates to orient individuals to new sensory conditions, investigating change-related brain activity may help us elucidate the mechanisms of preattentive activation processes in the brain in response to sensory changes. To study this neural change detection system, we have previously examined change-related cortical responses that were specifically evoked by a new sensory event (Tanaka et al., 2008, 2009a; Inui et al., 2010a, b, 2012, 2013, 2018; Nishihara et al., 2011; Takeuchi et al., 2017, 2018). Similar change-related responses are identified in the auditory system (Jones, 1991; Akiyama et al., 2011; Yamashiro et al., 2011a; Ohoyama et al., 2012; Otsuru et al., 2012; Nakagawa et al., 2014), visual system (Urakawa et al., 2010a, b), and tactile system (Yamashiro et al., 2008, 2009, 2011b; Otsuru et al., 2011; Kodaira et al., 2013). Because change-related responses depend on the magnitude of the change in the sensory stimulus (Inui et al., 2010a, b; Nishihara et al., 2011; Yamashiro et al., 2011a), the length of the preceding control stimulus being compared (Inui et al., 2010a; Akiyama et al., 2011; Yamashiro et al., 2011a), and the probability of the control and change stimulus (Inui et al., 2010b; Ohoyama et al., 2012), the generation of change-related responses is based on sensory memory and comparison processes. An individual’s change-related cortical response can be distinctly observed using electroencephalography (EEG) or magnetoencephalography (MEG) without the individual’s active involvement. Therefore, these are useful tools for investigating higher brain function. Although such EEG or MEG responses are anticipated to relate to faster reactions to new sensory events, it remains unclear whether change-related cortical responses reflect processing speed.

In the present study, we simultaneously recorded auditory steady-state response (ASSR) and change-related response to investigate acceleration effects of sensory change events. Steady-state responses (SSRs) are electrophysiological responses driven by a train of stimuli delivered at a sufficiently high rate, and ASSRs reportedly reach maximum amplitude at approximately 40 Hz (Galambos et al., 1981; Ross et al., 2000). Previous research using MEG (Herdman et al., 2003; Ross, 2008) and positron emission tomography (Pastor et al., 2002) has reported that ASSRs originate in primary auditory cortical areas. Although the generation mechanisms of ASSRs have not yet been completely elucidated (Presacco et al., 2010), there are two main interpretations: (i) ASSRs are the superposition of auditory middle latency response components (Plourde et al., 1991; Galambos and Makeig, 1992a, b; Suzuki et al., 1994; Bohórquez and Ozdamar, 2008) and (ii) ASSRs relate to oscillatory gamma band activity representing auditory object representation (Santarelli et al., 1995; Santarelli and Conti, 1999; Ross et al., 2002, 2005). The effect of a salient sensory stimulus on an SSR is known as phase resetting, and it induces the modulation of the amplitude and phase of the SSR. Rohrbaugh et al. (1989, 1990a,b) examined the effects of a foreground auditory or visual stimulus on 40-Hz ASSR evoked by a background rhythmic probe stimulus and observed a reduction of both the amplitude and latency of ASSR. Makeig and Galambos (1989) reported that similar phase resetting occurred in 40-Hz ASSR with sudden changes in the frequency or intensity of the train of stimuli. In a study using an auditory oddball paradigm, button pressing in response to a rare stimulus caused phase resetting in 40-Hz ASSR (Rockstroh et al., 1996). Ross et al. investigated phase resetting in greater detail, revealing that ASSR was modulated by stimulus onset (Ross et al., 2002), changes in periodicity of the sound stimulus (Ross and Pantev, 2004), and presence of an interfering stimulus (Ross et al., 2005). They suggested that such perturbing stimuli reset the oscillations and shift back the ASSR phase to that of the driving source (Ross et al., 2005).

In the present study, we aimed to investigate whether the ASSR phase resetting—particularly its temporal aspect—was influenced by the probability of the perturbing sound stimulus under an oddball paradigm. Because of its steepness, ASSR is superior to the middle latency components of auditory-evoked magnetic fields (AEFs) for observing subtle changes in processing timing. In our recent study, the acceleration of sensory processing was distinctly observed using the tactile-evoked steep transient N20 (Sugiyama et al., 2018). MEG can clearly record ASSR at the millisecond range, rendering it a useful approach for investigating the effects of acceleration on ASSR. We hypothesized that ASSR is affected by physical sound changes as well as the probability of sound changes, indicating that ASSR depends on sensory memory and comparison processes, similar to change-related responses. Furthermore, we believed that ASSR might be useful for elucidating the processing speed that could not be clarified by studies using change-related responses. In a study on change-related responses, the latency of the response to a rare stimulus tended to be shorter than that to a frequent stimulus (Ohoyama et al., 2012). Using ASSR, we anticipated that the effect of the stimulus probability on the processing speed would be clearly shown as the latency shift due to phase resetting.

## Materials and Methods

### Ethics Statement

This study was approved by the Ethics Committee of the National Institute for Physiological Sciences, Okazaki, Japan, and was conducted in accordance with the Declaration of Helsinki. Written informed consent was obtained from all the participants before experimentation.

### Subjects

A total of 13 healthy volunteers (four females and nine males) aged 21–54 years (mean, 30.4 ± 9.2) participated in the present study. The participants had no history of mental or neurological disorders or substance abuse in the last 2 years and were not taking any medication at the time of testing. All participants exhibited a hearing threshold <30 dB at 1,000 Hz, as assessed by an audiometer (AA-71, RION, Tokyo, Japan).

### Auditory Stimulation

**FIGURE 1:**
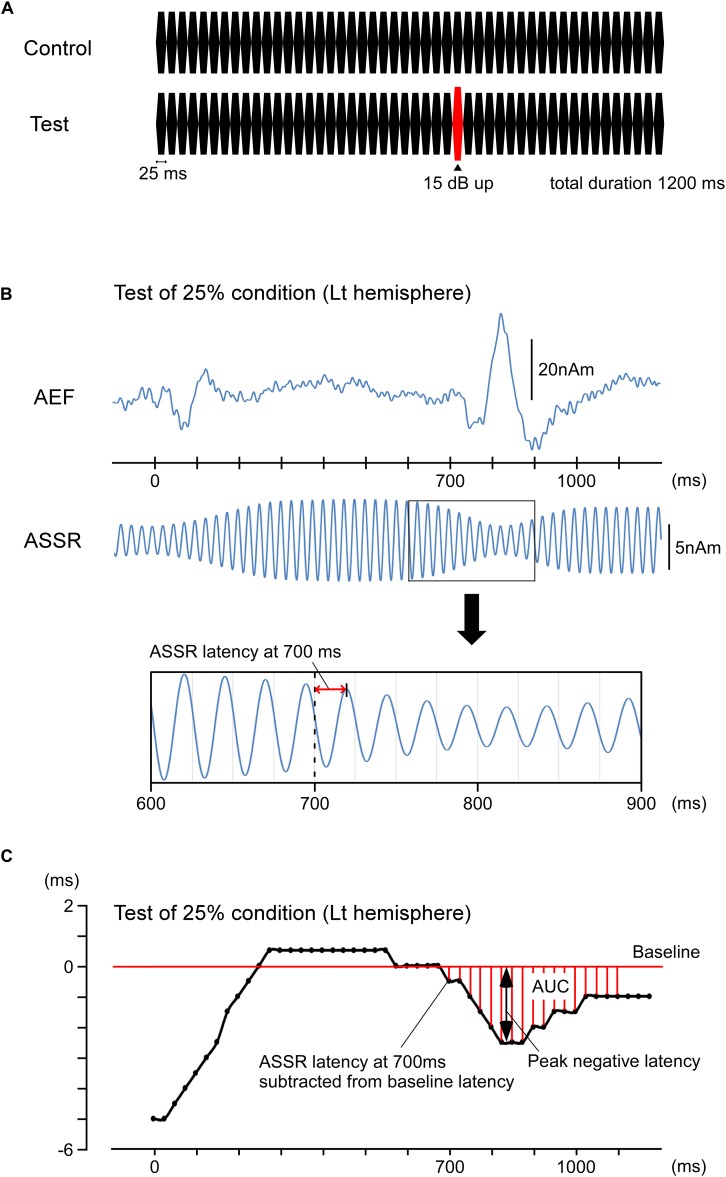
Methods for calculating the phase shift. Data for the left hemisphere of a representative participant to the test stimulus under an oddball paradigm with the 75% control stimuli and 25% test stimuli. **(A)** Stimulation paradigm. There were four different probabilities of the test stimulus—0, 25, 75, and 100%. **(B)** The upper trace shows the source strength waveforms of auditory-evoked magnetic fields (AEFs). The lower trace shows the source strength waveforms of ASSR. The latency of ASSR at 700 ms was defined as the interval to the peak of the first upward wave after 700 ms. The ASSR latencies at 600, 625, 650, and 675 ms were similarly obtained, averaged, and used as the standard. The ASSR latencies for all sampling points were expressed as the deviation from the standard. **(C)** The peak latency of ASSR sine waves is plotted for each time point. The peak negative latency and area under the baseline curve (AUC) are shown in the figure.

Repeats of a pure tone were used as auditory stimuli. The pure tone was 800 Hz in frequency, 70 dB in sound pressure level (SPL), and 25 ms in duration (rise/fall, 5 ms). The oddball paradigm comprised a control stimulus of a train of 48 pure tones (total duration, 1,200 ms) and a test stimulus of a similar train of 48 pure tones in which the 29th tone at 700 ms was increased by 15 dB (Figure 1A). Therefore, the present interest was the effects of the 29th sound on auditory-evoked responses. The sound stimulus was presented binaurally via earpieces (E-A-RTONE 3A, Aero Company, Indianapolis, IN, United States), and the sound pressure was controlled by an audiometer (AA-71, RION, Tokyo, Japan).

### MEG Recordings

Magnetic signals were recorded using a 306-channel whole-head MEG system (Vector-view, Elekta Neuromag, Helsinki, Finland) comprising 102 identical triple sensor elements. Each sensor element included two orthogonal planar gradiometers and one magnetometer coupled with a multi-superconducting quantum interference device, thereby providing three independent measurements of the magnetic fields. MEG signals were recorded using 204 planar-type gradiometers that were sufficiently powerful to detect the largest signal over local cerebral sources. Before recording, a current was fed to four head position indicator (HPI) coils placed at known sites for obtaining the exact location of the head with respect to the sensors, and the resulting magnetic fields were measured using the magnetometer; this approach allowed for aligning the individual head coordinate system with the magnetometer coordinate system. The four HPI coils attached on the individual’s head were measured with respect to the three anatomical landmarks using a 3D digitizer. The *X*-axis was fixed with the preauricular points, with right being the positive direction. The positive *Y*-axis passed via the nasion, and the *Z*-axis pointed upward. Signals were recorded using a band-pass filter of 0.1–300 Hz and digitized at 4,000 Hz. Epochs with MEG signals of >2.7 pT/cm were excluded from the averaging. The waveform was digitally filtered with a band-pass filter of 37.5–42.5 Hz when we focused on the 40-Hz SSRs and was otherwise filtered with a band-pass filter of 1–200 Hz and notch filter of 37.5–42.5 Hz.

### Procedure

Experiments were performed in a quiet, magnetically shielded room. Throughout the experiment, participants sat in a chair and watched a silent movie on a screen placed 1.5 m in front of them. To investigate the effects of the probability of a sudden change in sound pressure under an oddball paradigm on ASSR, we used four different probabilities of the test stimulus, namely 0, 25, 75, and 100%. Throughout the manuscript, the probability of a condition described is that for the test stimulus. The MEG signals for each condition were recorded in different blocks. To minimize order effects, approximately half of trials per condition were performed in a block, and the order of the eight blocks was randomized among participants. In the 25 and 75% probability conditions, the control and test stimulus were randomly presented. The intertrial interval, i.e., the stimulus onset asynchrony, was 1,500 ms. Analysis was conducted from 100 ms before to 1,200 ms after the onset of auditory stimulation. A minimum of 100 artifact-free epochs were averaged for the cortical responses to each stimulus per participant.

### Analysis

The Brain Electrical Source Analysis software package (BESA GmbH, Germany) was used to perform dipole analyses. First, AEFs were analyzed. Under a band-pass filter of 1–200 Hz and notch filter of 37.5–42.5 Hz, the MEG waveforms for the test stimulus under the three conditions of 25, 75, and 100% were combined. The equivalent current dipole for the main component of N100m was estimated for each hemisphere as described previously (Inui et al., 2006). The two-dipole model thus obtained was applied to the MEG signals for the three abovementioned conditions. The test stimulus with a sudden sound pressure increase at 700 ms evoked a change-related response with peaks at approximately 765 (P50) and 815 (N100) ms, and we measured the peak amplitudes in time windows of 750–780 and 780–850 ms, respectively, using the source strength waveforms. Peak-to-peak amplitudes were calculated for P50m–N100m response and compared across conditions using two-way repeated-measures analysis of variance (ANOVA) with the probability of the test stimulus and hemisphere as independent variables. The hemisphere was included in independent variables because there have been some previous studies showing right hemisphere predominance for change-related responses (Inui et al., 2010b, 2012, 2013). To assess differences between conditions, *post hoc* multiple comparisons were performed using Bonferroni-adjusted *t*-tests. All statistical analyses were performed with the level of significance set at 0.05.

Further, the 40-Hz ASSR was analyzed. Under a band-pass filter of 37.5–42.5 Hz, the MEG waveforms of all conditions were combined. The equivalent current dipole for the main component of ASSR was then estimated per hemisphere in a time window of 300–700 ms. The goodness-of-fit value of all participants was 92.4 ± 4.2% using the two-dipole model. The obtained two-dipole model was applied to the MEG signals from all conditions. Using the source strength waveform, the peak of each 40-Hz wave was measured. We defined the peak of the first upward wave (anterior-directing intracellular current) after 700 ms as the latency at “700 ms” (Figure 1B) and measured the peak latencies of ASSR from 0 to 1,150 ms at 25-ms intervals. The measured latencies were subtracted from each latency point, and the baseline was adjusted by the mean latency at 600, 625, 650, and 675 ms. Because a sudden change in sound pressure hastened the ASSR phase, the peak latency of a wave after the change in sound pressure was typically negative relative to baseline. Therefore, a greater reduction of peak latency indicated a greater effect. For comparisons among conditions, the minimum or peak negative latency after 700 ms and the area under the baseline curve (AUC) from 700 to 1,100 ms were used (Figure 1C). Both analyses were conducted using two-way repeated-measures ANOVA with the probability of the test stimulus and hemisphere as independent variables. To assess differences between conditions, *post hoc* multiple comparisons were performed using Bonferroni-adjusted *t*-tests. All statistical analyses were performed with the level of significance set at 0.05.

## Results

**FIGURE 2:**
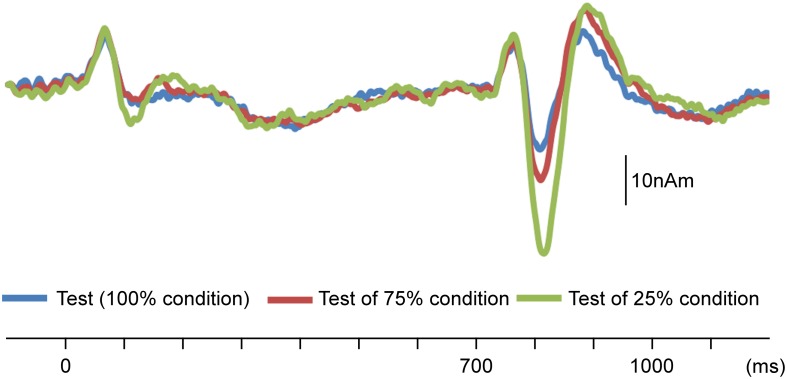
Effects of probability on change-related cortical responses. Grand-averaged waveforms of auditory-evoked magnetic fields (AEFs) following the test stimulus across 13 participants are shown. Probability refers to that for the test stimulus.

In the initial analysis of AEFs, the sudden increase in sound pressure elicited clear triphasic responses. Figure 2 shows the grand-averaged waveforms. The results of the two-way ANOVA (Probability × Hemisphere) showed that the probability of the test stimulus significantly affected the N100m amplitude (*F*_2__,__11_ = 28.08; *p* = 4.8 × 10^–5^; partial η^2^ = 0.84), whereas the hemisphere did not (*F*_1__,__12_ = 0.24; *p* = 0.63; η^2^ = 0.02). *Post hoc* tests revealed that the N100m amplitude was significantly higher when the probability of the test stimulus was lower (*p* < 0.017; Table 1).

**TABLE 1:** Mean amplitude of N100m for the test stimulus under the three probability conditions.

**Condition**	**Amplitude (nAm)**
Test (100% condition)	26.86 (9.07)
Test of 75% condition	32.17 (10.06)
Test of 25% condition	49.78 (17.15)

*Data are shown as mean (SD) values.*

**FIGURE 3:**
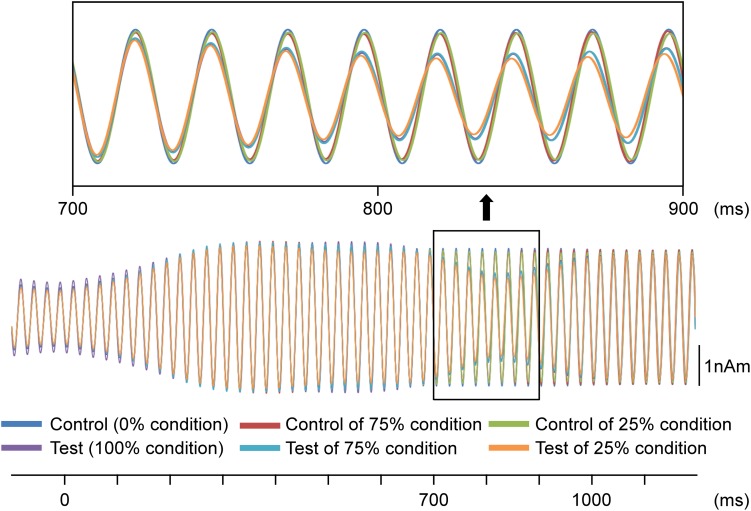
Effects of the probability on auditory steady-state response (ASSR). Grand-averaged waveforms across 13 participants are shown. Probability refers to that for the test stimulus.

**FIGURE 4:**
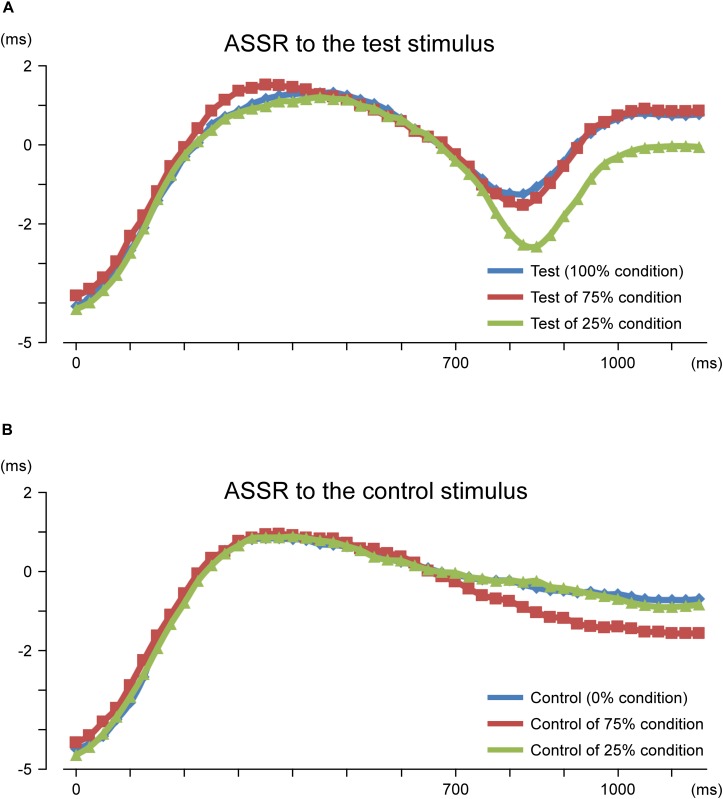
Time course of the peak latency of 40-Hz auditory steady-state response (ASSR). Mean peak latency of each ASSR sine wave is plotted for each time point. Results for ASSR to the test stimulus **(A)** and control stimulus **(B)** are shown. Probability refers to that for the test stimulus.

In the second analysis, the train of pure tones elicited clear sine waves. Figure 3 shows the grand-averaged waveforms for all conditions. As shown, the test stimulus shifted the ASSR phase regardless of the probability. Figure 4A presents a comparison of the effects of the test stimulus under the three probability conditions and shows that the phase shift was greater for the 25, 75, and 100% conditions in that order (Table 2). The two-way ANOVA showed a significant main effect for probability of the test stimulus (*F*_2__,__11_ = 10.05; *p* = 0.003; η^2^ = 0.65) but not for the hemisphere (*F*_1__,__12_ = 0.57; *p* = 0.47; η^2^ = 0.05). *Post hoc* tests revealed that the latency for the 25% condition was significantly shorter than that for the 100% (*p* = 0.008) and 75% (*p* = 0.002) conditions; however, there was no significant difference between the latter two probabilities (*p* > 0.99). Concerning the AUC from 700 to 1,100 ms, the two-way ANOVA indicated that probability was a significant factor (*F*_2__,__11_ = 8.59; *p* = 0.006; η^2^ = 0.61), whereas the hemisphere was not (*F*_1__,__12_ = 0.94; *p* = 0.35; η^2^ = 0.07). *Post hoc* tests revealed that the area for the 25% condition was significantly larger than that for the 75 and 100% conditions (*p* < 0.017); however, there was no significant difference between the areas for the latter two conditions (*p* > 0.99; Table 3).

**TABLE 2:** Mean latency shift of auditory steady state response for all conditions.

**Condition**	**750 ms**	**775 ms**	**800 ms**	**825 ms**	**850 ms**	**875 ms**	**900 ms**	**925 ms**
Test (100% condition)	1.09 (0.79)	1.35 (0.85)	1.42 (0.98)	1.44 (1.03)	1.27 (1.15)	1.02 (1.23)	0.69 (1.34)	0.25 (1.45)
Test of 75% condition	1.23 (0.87)	1.44 (0.97)	1.63 (1.06)	1.71 (1.12)	1.53 (1.09)	1.19 (1.02)	0.78 (1.03)	0.36 (1.01)
Test of 25% condition	1.35 (1.00)	1.89 (1.25)	2.35 (1.41)	2.64 (1.47)	2.68 (1.56)	2.41 (1.47)	1.97 (1.41)	1.56 (1.33)
Control (0% condition)	0.30 (0.46)	0.32 (0.50)	0.32 (0.54)	0.42 (0.60)	0.50 (0.63)	0.55 (0.64)	0.57 (0.64)	0.61 (0.62)
Control of 75% condition	0.68 (0.69)	0.75 (0.69)	0.81 (0.67)	0.97 (0.74)	1.08 (0.75)	1.20 (0.74)	1.22 (0.80)	1.35 (0.80)
Control of 25% condition	0.27 (0.43)	0.33 (0.47)	0.31 (0.60)	0.35 (0.69)	0.33 (0.73)	0.47 (0.76)	0.50 (0.79)	0.56 (0.84)

*Data are shown as mean (SD) values.*

**TABLE 3:** Mean area under the baseline curve (AUC) from 700 to 1,100 ms for all conditions.

**Condition**	**AUC**
Test (100% condition)	13.35 (7.60)
Test of 75% condition	13.46 (8.65)
Test of 25% condition	25.06 (13.78)
Control (0% condition)	10.14 (6.64)
Control of 75% condition	19.47 (9.55)
Control of 25% condition	10.79 (6.77)

*Data are shown as mean (SD) values.*

The effects of the control stimulus were then assessed. Figure 4B presents a comparison among the three probability conditions. Regarding minimum latency, the two-way ANOVA showed a significant main effect for probability (*F*_2__,__11_ = 10.31; *p* = 0.003; η^2^ = 0.65) but not for hemisphere (*F*_1__,__12_ = 0.90; *p* = 0.36; η^2^ = 0.07). *Post hoc* tests revealed that the latency for the 75% condition was significantly shorter than that for the control-alone (*p* = 0.003) and 25% (*p* = 0.003) conditions; however, there was no significant difference between the latter two conditions (*p* > 0.99). The ASSR latency for the 75% condition was shorter than that for the control-alone condition by an average of 0.78 ms. Similarly, probability was a significant factor for determining the AUC (*F*_2__,__11_ = 19.46; *p* = 2.5 × 10^–4^; η^2^ = 0.78), whereas hemisphere was not (*F*_1__,__12_ = 1.09; *p* = 0.32; η^2^ = 0.08). *Post hoc* tests demonstrated that the AUC for the 75% condition was significantly larger than those for the control-alone and 25% conditions (*p* < 0.005), whereas the AUC between the latter two conditions was not significantly different (*p* > 0.99).

## Discussion

The present study examined the effect of the probability of the test and control stimuli under an oddball paradigm on change-related cortical responses and ASSR. As the probability decreased, i.e., the strength of the memory trace for the control stimulus to be compared increased, the amplitude of the P50m–N100m response to the test stimulus increased, confirming the results of a previous study (Ohoyama et al., 2012). This finding indicated that change-related cortical responses do not depend solely on physical change. Instead, ASSR was modulated in a similar manner by sound onset and a sudden change in sound pressure. The observed features of phase resetting are consistent with those reported in previous studies (Makeig and Galambos, 1989; Rohrbaugh et al., 1989, 1990a,b). Specifically, the latency of ASSR was reduced by sound onset as well as changes in sound pressure approximately 300 ms after event onset. The latency shift from the baseline of the 100% condition (test alone) was approximately 4 ms for sound onset and 1.4 ms for change in sound pressure, which are consistent with the findings of previous reports (Ross et al., 2002, 2005; Ross and Pantev, 2004). Moreover, these findings are in agreement with the fact that onset response is a type of change-related response (Nishihara et al., 2011). As for the effect of the probability of the test stimulus, the latency shift of ASSR for the 25% condition was significantly larger than that for the 75 and 100% conditions, indicating that ASSR and change-related cortical response depend on physical changes as well as on sensory memory and comparison processes (Inui et al., 2010b; Ohoyama et al., 2012).

The notion that the ASSR phase shift is a higher brain function was further supported by the results for the control condition in which there were no sensory changes. Compared with the control-alone and 25% conditions, a significantly greater latency shift was observed for the 75% condition. In other words, regardless of whether the control or test stimulus was used, the effect of acceleration on ASSR was observed when the stimulus was rare. This indicates that ASSR is modulated without changes in peripheral inputs and that brain areas higher than the primary cortex could be involved in exerting acceleration effects.

**FIGURE 5:**
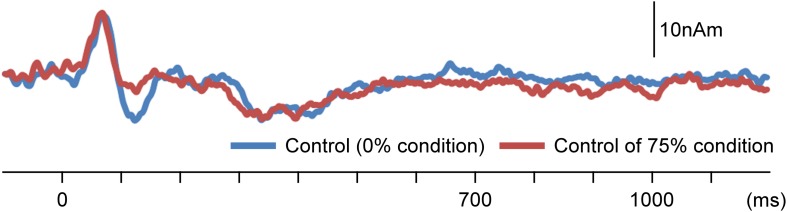
Comparison of auditory-evoked magnetic fields (AEFs) in response to control stimulus between 0% (control alone) and 75% (control 25%) conditions. Grand-averaged waveforms across 13 participants are shown. Probability refers to that for the test stimulus.

Ross (2008) stated that a sensitivity of 40-Hz ASSR to stimulus changes might be advantageous compared with conventional AEFs for clinical applications and neuroscience research. In the present study, the rare control stimulus had a significant effect on ASSR but showed no effect on AEFs (Figure 5), supporting the notion that ASSR is a more sensitive measure than AEFs for observing brain responses to subtle sensory changes. The lack of change-related response in AEFs to the rare control stimulus might be because of the high probability of the rare control stimulus under the oddball paradigm. In the present study, we adopted a ratio of 1:3 (25% rare control stimulus) to shorten the measurement time. If the rare control stimulus had been presented at a lower probability, change-related AEF responses might have been observed.

Because it reliably measures peak latency, ASSR is superior to AEF for providing information on processing speed. The present ASSR results provide evidence of accelerated sensory processing when a new event occurs, which is consistent with the idea that change-driven brain responses are used for orientation. Considering that change-related response is a defense reaction (Inui et al., 2012), it should enhance processes for enacting appropriate behaviors. In our previous studies on change-related responses, the latency decreased with an increase in the magnitude of change in sound properties (Inui et al., 2010a, b; Nishihara et al., 2011). Jaskowski et al. (1994) showed that both reaction time and evoked potential latency decreased with an increase in stimulus intensity. Therefore, these findings indicate that both change-related cortical response and reduction in the latency of ASSR may lead to sensory facilitation. In the present study, importantly, the reduction in the latency of ASSR clearly indicated that a new sensory event increased the rate of ongoing sensory processing. Furthermore, ASSR reflects an endogenous response rather than a simple reaction to a physical sensory input as shown in the present result. This finding is consistent with the notion by Ross (2008) that ASSR reflects an internal stimulus representation. Therefore, ASSR is considered a good indicator of an individual’s inherent responsiveness to sensory changes. We believe that the present study that focuses on the latency of ASSR will help clarify the processing speed of the neural change detection system.

**FIGURE 6:**
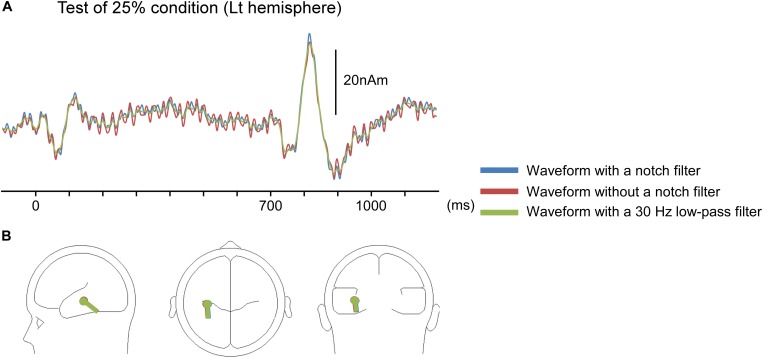
Impact of a notch filter on auditory-evoked magnetic fields (AEFs). Data for the left hemisphere of a representative participant to the test stimulus under an oddball paradigm with the 75% control stimuli and 25% test stimuli. **(A)** The source strength waveforms of AEFs with a notch filter of 37.5–42.5 Hz, without a notch filter, and with a 30-Hz low-pass filter. The response owing to auditory steady-state response (ASSR) was attenuated by the notch filter. **(B)** Dipole location for waveforms of each filter condition.

There are some limitations in the present study. Although we analyzed AEFs under the notch filter of 37.5–42.5 Hz to remove 40-Hz ASSR, other frequency responses affect the 40-Hz ASSR (e.g., Ross et al., 2003). In fact, as shown in Figure 6A, ASSRs could be slightly observed even after filtering. Although results of our preliminary study showed that effects on dipole location of the filtering was modest (Figure 6B), waveforms as well as the dipole location might have been affected by ASSR.

## Conclusion

Using a change-related paradigm, we revealed, for the first time to the best of our knowledge, that the latency of ASSR could be accelerated without changes in peripheral inputs, suggesting that brain areas higher than the primary cortex could be involved in acceleration effects and that faster processing in ASSR could contribute to shorter reaction times. Changes in the latency of ASSR could be a sensitive index of accelerated processing.

## Data Availability Statement

All datasets generated for this study are included in the manuscript/supplementary files.

## Ethics Statement

This studies involving human participants were reviewed and approved by Ethics Committee of the National Institute for Physiological Sciences, Okazaki, Japan. The patients/participants provided their written informed consent to participate in this study.

## Author Contributions

SS and KI designed the work, analyzed the data, and drafted the manuscript. SS, TK, NT, MN, and KI performed the experiments. TS commented on the manuscript. All authors read and approved the manuscript.

## Conflict of Interest

The authors declare that the research was conducted in the absence of any commercial or financial relationships that could be construed as a potential conflict of interest.

## Abbreviations

AEF: auditory-evoked magnetic fields
ASSR: auditory steady-state response
AUC: area under the baseline curve
EEG: electroencephalography
MEG: magnetoencephalography
